# Establishment methods and research progress of livestock and poultry immortalized cell lines: A review

**DOI:** 10.3389/fvets.2022.956357

**Published:** 2022-09-02

**Authors:** Dongxue Guo, Li Zhang, Xiaotong Wang, Jiahui Zheng, Shudai Lin

**Affiliations:** College of Coastal Agricultural Sciences, Guangdong Ocean University, Zhanjiang, China

**Keywords:** livestock and poultry, immortalization, cell line, methods, telomerase activity

## Abstract

An infinite cell line is one of the most favored experimental tools and plays an irreplaceable role in cell-based biological research. Primary cells from normal animal tissues undergo a limited number of divisions and subcultures *in vitro* before they enter senescence and die. On the contrary, an infinite cell line is a population of non-senescent cells that could proliferate indefinitely *in vitro* under the stimulation of external factors such as physicochemical stimulation, virus infection, or transfer of immortality genes. Cell immortalization is the basis for establishing an infinite cell line, and previous studies have found that methods to obtain immortalized cells mainly included physical and chemical stimulations, heterologous expression of viral oncogenes, increased telomerase activity, and spontaneous formation. However, some immortalized cells do not necessarily proliferate permanently even though they can extend their lifespan compared with primary cells. An infinite cell line not only avoids the complicated process of collecting primary cell, it also provides a convenient and reliable tool for studying scientific problems in biology. At present, how to establish a stable infinite cell line to maximize the proliferation of cells while maintaining the normal function of cells is a hot issue in the biological community. This review briefly introduces the methods of cell immortalization, discusses the related progress of establishing immortalized cell lines in livestock and poultry, and compares the characteristics of several methods, hoping to provide some ideas for generating new immortalized cell lines.

## Introduction

As the basic structural and functional unit of life activities, cells are widely used as experimental tools in various studies, especially in the fields of molecular biology and biomedical research. Currently, there are two types of animal cells commonly used in laboratories: primary cells and infinite cell lines (1). Primary cells refer to cells that are directly collected from organism tissues and cultured in a simulated *in vivo* environment (2). Most of them are collected from tissues of experimental animals such as mice and rabbits, and chicken embryos (3, 4). Take myoblast cells as an example to briefly describe the general process for collecting adherent cells. First, collect fresh muscle tissue samples from a slaughterhouse and transport them to a cell culture laboratory under sterile conditions (5). Small-sized experimental animals such as chicken embryos, whose muscle tissue can also be separated directly on the laboratory sterile bench (6). Then, wash the muscle tissue with 70% ethanol or 1 × phosphate buffered saline (PBS) containing 1% penicillin-streptomycin to remove surface dirt, and cut it into small pieces. Obtain the suspension containing myoblasts after mechanical dispersion and enzymatic digestion (commonly used are 0.1% collagenase and 0.25% trypsin solutions) (7, 8). Finally, remove tissue and cell debris in the suspension using a 40-μM cell strainer and perform low-speed centrifugation to collect primary myoblast cells (9–12). It is worth mentioning that the collected primary cells are suspended in a complete medium supplemented with an appropriate amount of fetal bovine serum (FBS) and cultured in monolayers at 37°C in a humidified atmosphere containing 5% carbon dioxide (simulating the environment in which cells survive and replicate *in vivo*) (13, 14).

The collected primary cells are almost identical to their source cells in morphology and characteristics. However, their ability to rapidly proliferate and differentiate *in vitro* is limited (15, 16). Even primary tumor-derived cells cannot continue to proliferate after a certain number of passages *in vitro* (17). In contrast, an infinite cell line is a population of non-senescent cells that escape cell cycle restriction and can proliferate indefinitely *in vitro* (18). In other words, achieving cell immortality is the basis for establishing infinite cell lines. Cell immortalization is one of the hotspots in biological research. It refers to the process of making cells cultured *in vitro* escape the senescence period of cell proliferation under the influence of external factors to obtain the ability of infinite division (1). Previous research has revealed that telomeres and telomerase activity were closely related to cell immortalization (19). Telomeres, special DNA-protein complexes presenting at the ends of eukaryotic chromosomes, are comprised of simple repetitive and highly conserved DNA sequences with guanine (G) base-rich and related proteins. They are involved in DNA replication and play important roles in maintaining a stable and complete replication of chromosomes (20). Along with proliferation and division of cells from normal animal tissues (nerve tissue, muscle tissue, etc.), telomeres get shortened, and cell proliferation will be inhibited to enter the senescence period. At this time, if the activity of telomerase is extremely low, the cell will reach the crisis stage and finally enter apoptosis under gene regulation. On the contrary, immortalized cells or tumor cells can maintain constant telomere length because of the activation of telomerase (21). In addition, the expression of tumor suppressor gene *p53* or *Rb* is also an important regulatory point in the process of cell immortalization (22, 23).

Cell lines bypass ethical issues associated with the use of animal and human tissues, providing an endless supply of a homogeneous cellular material that is cost-effective and very convenient to use. In addition, a cell line avoids collection of animal tissues from slaughterhouses, reducing the risk of endogenous contamination (24). Previous studies have suggested that many established immortalized cell lines could maintain the shape, characteristics, and functions of primary cells, and replace primary cells to provide convenient and reliable experimental materials for basic scientific research studies, clinical treatments, bioengineering pharmaceuticals, and vaccine research and development (25–27). However, some immortalized cells do not proliferate permanently despite their extended lifespan compared with primary cells (28). After multiple population doublings (PDs), cells will gradually senesce and loss important genetic characteristics (15, 18). Therefore, we summarized the established livestock and poultry cell lines and compared different methods to generate a stable infinite cell line hoping to find a better way to maximize the PDs of cells while maintaining their normal functions.

## Methods for obtaining immortalized cells

**Figure 1:**
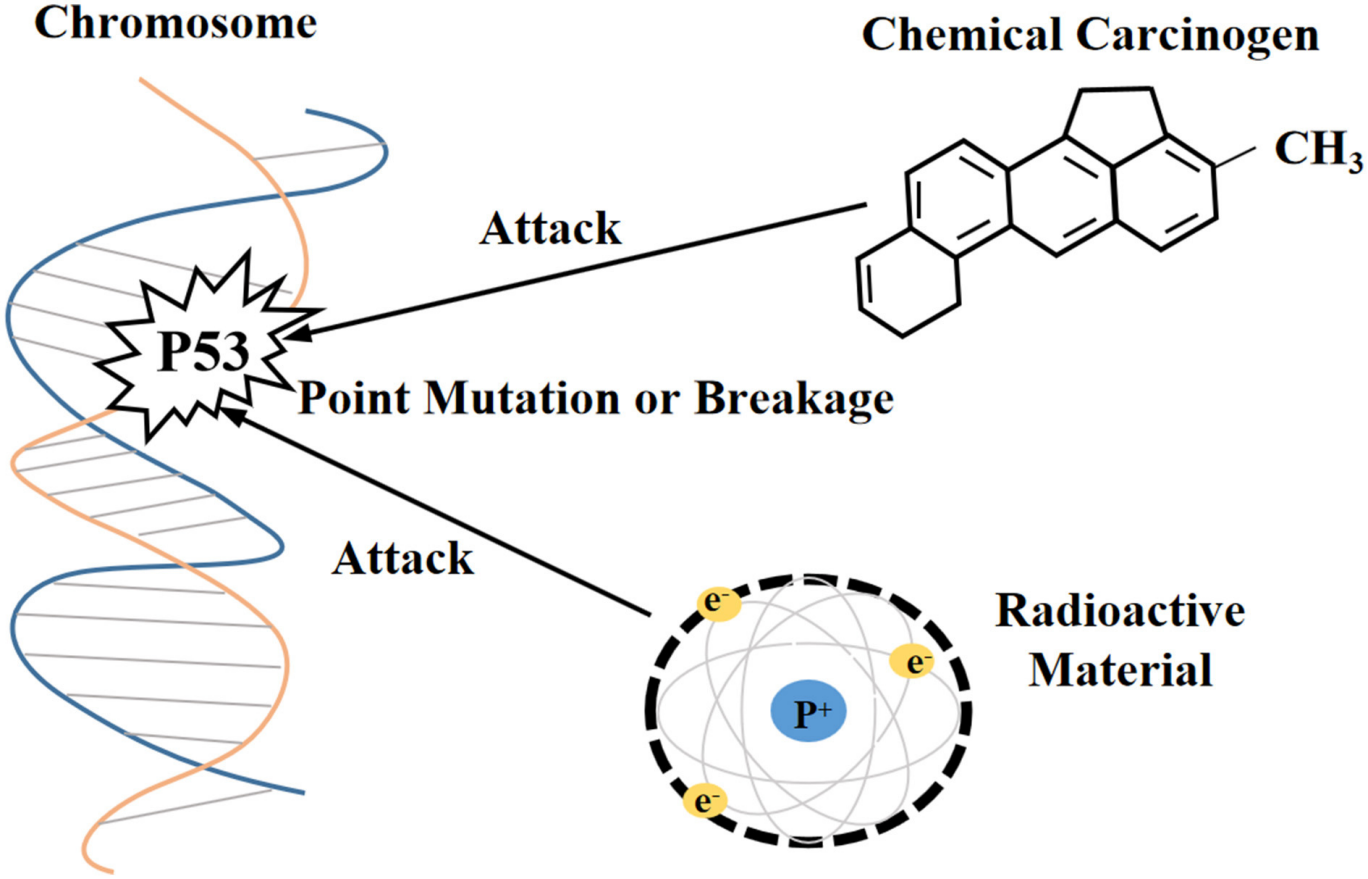
Physicochemical stimulation disrupts the molecular structure of proto-oncogenes and tumor suppressor genes.

**Figure 2:**
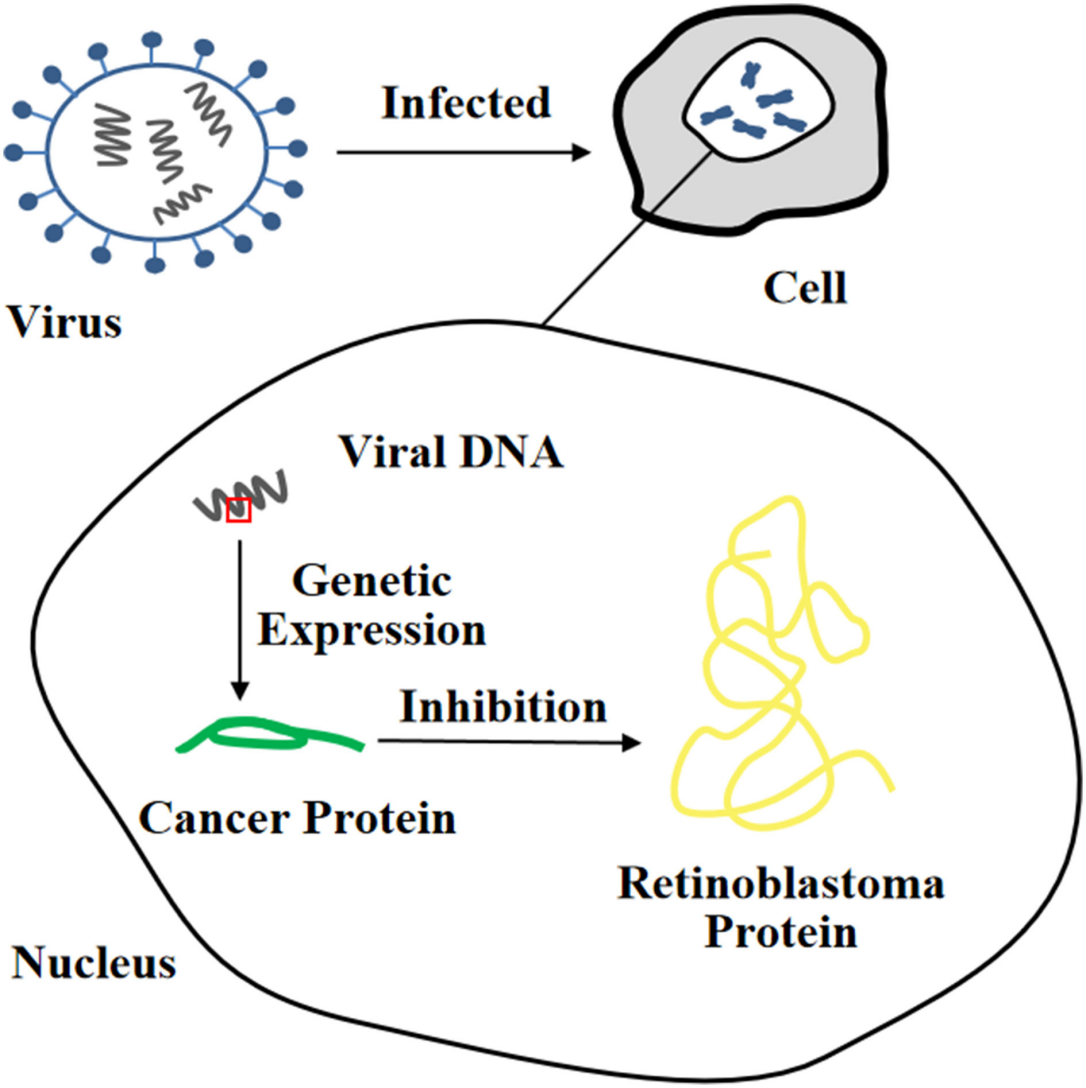
Heterologous expression of viral oncogene inhibits the function of tumor suppressor protein.

**Figure 3:**
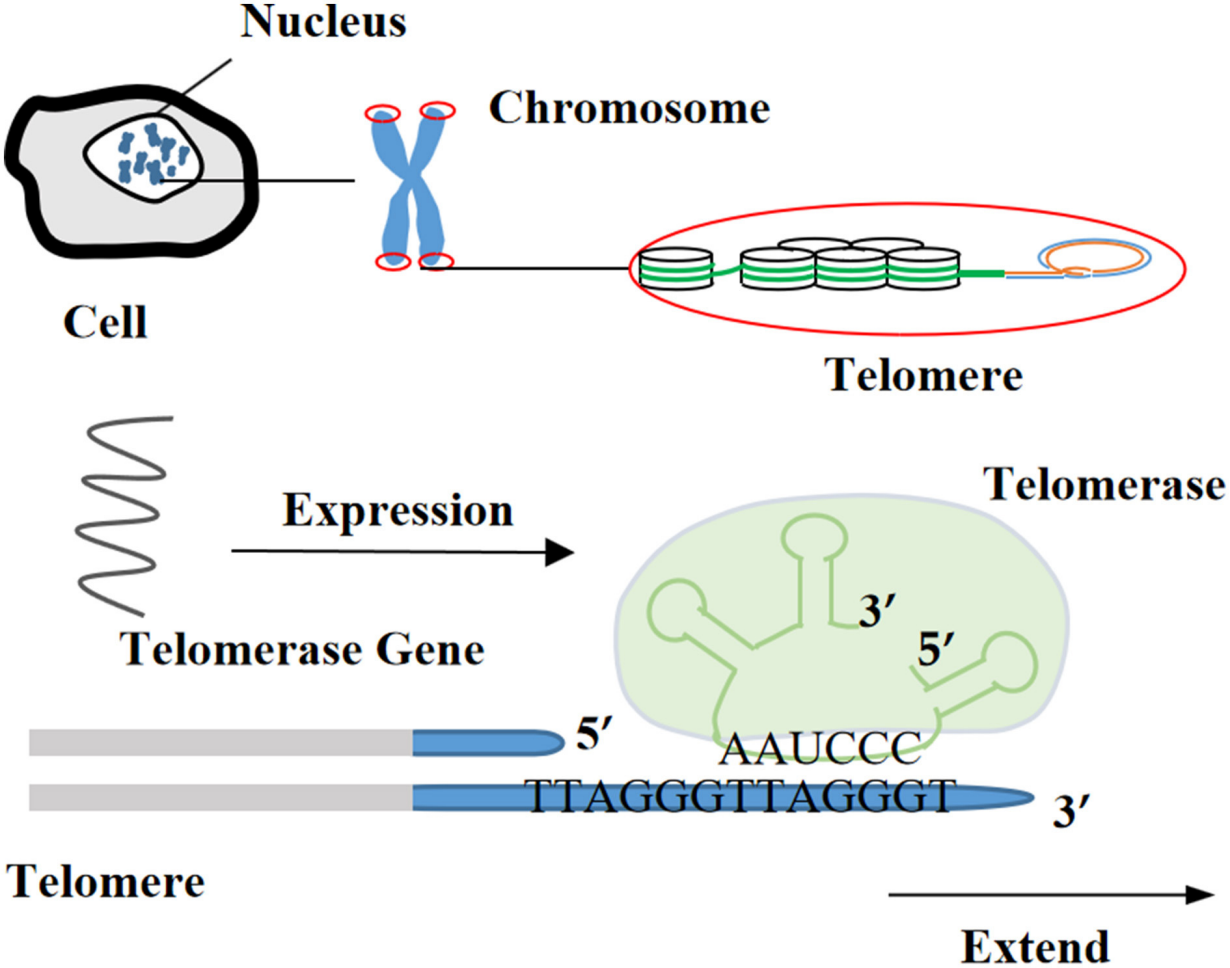
Mechanism of expressing telomerase reverse transcriptase gene.

Currently, the methods for obtaining the immortalization of human and animal cells are mainly divided into four categories (1, 29): (i) destroying the regulation of proto-oncogenes or tumor suppressor genes on the cell cycle through physical and chemical stimulation, which was a technique often utilized in early research (Figure 1), (ii) inducing the heterologous expression of viral oncogenes to help cells escape the cell cycle control (Figure 2), (iii) stimulating the activity of cellular telomerase to overcome the replicative senescence caused by telomere shortening and realize the infinite proliferation of cells *in vitro* (Figure 3), and (iv) spontaneous formation.

### Physical and chemical stimulation

#### Immortalization of cells induced by radioactive factors

In previous studies, researchers have attempted to induce cells with unlimited proliferation using X-rays or gamma rays. For example, results from an experiment indicated that human skin fibroblasts with a mutant *p53* allele could proliferate continually and exceeded 450 PDs *in vitro* after periodic X-ray irradiation, whereas the unirradiated control group cells could only be cultured to 37 PDs (30). Relevant phenotypes of immortalized cells obtained with such methods could be transferred by DNA transfection, which has been demonstrated in mouse cells (31). Previous study has shown that in place of it was suggested that treatment with Harvery murine sarcoma virus (Ha-MSV) alone did not promote the transformation of normal human fibroblasts into immortalized or tumorigenic cells, while immortalized fibroblasts KMST-6 formed by Co60γ-ray irradiation after treatment of Ha-MSV, and transplanted them into nude mice could acquire anchorage independent growth potential and eventually generated tumors (32). Therefore, radioactive factor-induced immortalized cells may increase the risk of tumorigenesis.

#### Immortalization of cells induced by chemical carcinogens

N-methyl-N-nitro-N-nitrosoguanidine (MNNG) (33) and 3-methylcholanthrene (34) are chemical carcinogens that induce cell immortalization. A previous study has observed that rabbit tracheal epithelial cells proliferated exponentially in the second week of culture and reached plateau in the third week. However, after experiencing the MNNG process, some rabbit tracheal epithelial cells showed a relative delay in the onset of proliferation and recovered clonal activity in a later stage of the plateau phase (35). Nevertheless, immortalized cells induced by chemical carcinogens do not necessarily retain normal morphology and are adhesion-dependent (33). Therefore, their carcinogenesis risk cannot be neglected.

### Heterologous expression of viral oncogenes

It is well-known that the simian virus 40 large T antigen (*SV40-LT*), human papilloma virus E6 or E7 protein (*HPV E6/E7*), and Epstein-Barr Virus (*EBV*) are oncogenes. Among them, the *SV40-LT* gene fragment is one of the most commonly used target fragments for inducing cell immortalization. Integrating it into the target cell nucleus for expression can cause inactivation of the p53 and Rb proteins, thereby changing cell proliferation activity and prolonging cell lifespan (36). However, the length of telomeres will gradually shorten until cells stop growing, and only a few cells can completely leave the cell cycle and continue to proliferate, eventually forming immortalized cell lines (37). In recent years, *SV40-LT* has been successfully used in the establishment of immortalized cell lines of livestock and poultry such as pigs (38), cattle (25), sheep (24), and ducks (39).

In addition, infection with *HPV E6/E7* can also immortalize a large number of different types of cells (40, 41). The HPV E6 protein, as one of the most common transforming proteins, can cause degradation of the p53 protein and upregulate the expression level of cellular-myelocytomatosis viral oncogene (*c-myc*) (42). Furthermore, it can also induce the expression of human telomerase reverse transcriptase (*hTERT*) and enable cells to acquire the ability of indefinite proliferation (43). There are many binding sites for c-myctranscription factor on the promoter of *hTERT*, so c-myc can mediate *hTERT* transcriptional activation and rapidly induce *hTERT* mRNA to express (44). The HPV E7 protein can lead to degradation of the Rb protein (45). It was reported that retroviruses containing the *HPV E6/E7* gene was used to infect human pancreatic duct epithelial cells to establish the corresponding immortalized cell line, which could be passaged more than 20 times, retaining the anchorage dependence of mammalian cells with non-carcinogenic effects (40). Currently, the EBV is mostly used to immortalize B lymphocytes. The EBV genome contains more than 100 genes, and only a few genes (so-called latent genes) can be expressed in EBV-infected B lymphocytes. For instance, it is capable of infecting B lymphoblastoid cells *in vitro*, activating the interaction of cytokines with their receptors by expressing latent proteins, and forming immortalized lymphoblastoid cell lines. It is worth noting that the most notable feature of immortalized B cells induced by *EBV* is increased telomerase activity (46).

### Telomerase causing cell immortalization

#### Telomerase

Telomerase is a kind of a specific reverse transcriptase and includes three components: telomerase RNA (TR), telomerase-associated protein, and telomerase reverse transcriptase (TERT) or telomerase catalytic subunit. Using its own RNA as a template to extend telomeres from the 3′-OH end of telomeric DNA or synthesize new telomeric DNA, it can compensate for the shortening of chromosome ends during cell division, so as to maintain the length of telomeres and prevent cells from the apoptosis caused by telomere depletion (47). Telomerase almost has no activity in normal cells but with expression in stem cells and germ cells. The activity of telomerase is elevated in most immortalized cell lines and various human tumor tissues, suggesting that telomerase activity is closely related to occurrence and development of tumors (48).

#### Rebuild telomerase activity to immortalize cells

In 1998, it was first reported that after the exogenous *hTERT* gene was introduced into telomerase-negative normal human retinal pigment epithelial cells, the intracellular telomerase was activated and the endogenous β-galactosidase (senescent marker) was significantly reduced (49). Besides, a previous study has claimed that after transfection with retrovirus-mediated exogenous *hTERT* gene, normal human breast epithelial cells gained stable telomere length, longer lifespan (40 PDs more than primary cells), less obvious β-galactosidase staining, and unchanged plasminogen activator inhibitor expression (PAI, another senescent marker) (50).

Furthermore, it has been determined that *hTERT* could improve telomerase activity, stabilize telomere length in cells, increase the number of cellular PDs, slow down cell senescence, and prolong the lifespan of culture *in vitro* (51–56). Certain cells can maintain their original morphology and function while obtaining the ability to proliferate indefinitely (57, 58). For example, immortalized human bone marrow mesenchymal stem cell line carrying *hTERT* has been subjected to 290 PDs without losing cell contact inhibitory function. By observing cell morphology at 95 and 275 PDs, it was found that transfected cells had the ability to transform into adipocytes, chondrocytes, and osteoblasts (59). Currently, *hTERT* transfection alone can immortalize many livestock and poultry cells (Table 1), or it can be combined with viral oncogenes to improve the success rate of obtaining immortalized cells (69).

**Table 1:** Immortalized livestock cell lines established by transfecting *hTERT* alone.

**Species**	**Cell line name**	**Cell line source**	**Immortality**	**References**
Swine	Fibroblast cell line	Primary fibroblasts prepared from pig ears, fetuses, and lung tissues	Cultured for 30–45 passages	(60)
	hTERT-POMECs	Primary porcine oral mucosal epithelial cells (POMECs) from the neonatal piglet.	Cultured for more than 150 passages *in vitro*	(61)
	iPMSCs	Fetal porcine pancreas mesenchymal stem cells	More than 80 passages	(62)
	EE cell line	Endocardial endothelium cells	Over 100 generations	(63)
	SUVECs	Umbilical vein endothelial cells	Passaged 50 times	(64)
Cattle	HTERT-AEC II	Type II alveolar epithelial cells	More than 50 passages	(4)
	hTERT-BME	Microvascular endothelial cells isolated from adrenal cortex	Over 80 passages	(65)
	BMET	Muscle epithelial cells	Cultured for 59 passages	(66)
Sheep	Fibroblast cell line	Lung fibroblasts	Cultured for about 120 days (50–80 PDS)	(67)
	hTERT-STCs	Primary trophoblast cells (STCs)	Cultured for 50 passages	(7)
	Microglia cell line	Brain macrophage	Passage up to 100 times	(26)
	Fibroblasts cell line	Fetal sheep fibroblasts	More than 180 PDs	(68)

### Spontaneously generated immortalized cells

During cell culture *in vitro*, some spontaneously immortalized cells are occasionally generated and show high proliferative potential without gene transfer (70–73). These cells achieve serum-independent growth and have higher saturation densities (74).

Rodent cells have a higher incidence of spontaneous immortalization, up to 10^−5^ or 10^−6^ (44). Previous research has discussed that human cells could escape aging only if both the *p53* and *Rb* genes were inactivated simultaneously, and that dysregulation of the ARF-p53 pathway alone in rodent cells was sufficient for eternal proliferation (75). By comparing the expression of multiple genes in early passage bovine mammary epithelial cells (bMECs), senescent bMECs, spontaneously immortalized bMECs (BME65Cs), and human breast cancer MCF-7 cell line (76), it was found that BME65Cs had the general features of normal BMECs in terms of morphology and karyotype etc., accompanied by endogenous *TERT* activity and telomeres stability. Compared with MCF-7 cells, the oncogene *c-myc* was only slightly upregulated in BME65Cs, and the breast tumor-related genes Bcl-2-associated athanogene 1 (*Bag-1*) and transcriptional repressor 1 (*TRPS-1*) were not detected. Likewise, the expression of tumor suppressor gene *p53* and cycle-dependent kinase inhibitory factor *p16INK4a* (also known as cyclin-dependent kinase inhibitor 2A, *CDKN2A*) in BME65Cs was decreased but not completely inactivated compared to earlier passages, indicating that spontaneous immortalized cell lines were not caused by mutations in the *p53* or *p16INK4a* gene. In addition, the expression level of DNA methyltransferase was upregulated, suggesting that the co-suppression of cell aging and mitochondrial apoptosis pathways orchestrated the immortalization process of BME65Cs (76). That means the mechanism by which spontaneously immortalized cells escape replicative senescence is poorly understood.

## Establishment and current status of livestock and poultry immortalized cell lines

### The common methods for establishing non-carcinogenic immortalized cell lines

It is well known that cancer cells also have the ability to proliferate indefinitely, and that cells may become cancerous during the process of establishing cell lines. Soft agar assay and nude mouse tumorigenesis assay are widely recognized methods for testing whether immortalized cell lines are tumorigenic (77, 78). Studies have found that immortalized cell lines induced by radioactive substances and chemical carcinogens may increase the formation of cancer cells, which are rarely used today (32, 33). Immortalized cell lines established by inducing the combined expression of immortality genes, proto-oncogenes, and cell cycle regulators are also tumorigenic, such as porcine pancreatic ductal epithelial cells, which are often used to generate tumor models (79, 80).

However, some immortalized cell lines can still avoid the generation of cancer cells while maintaining the morphological and physiological characteristics of primary cells (63, 81). The current common immortalization methods that do not cause any cancer growth are mainly by *hTERT* or *SV40-LT* expression induction, such as porcine oral mucosal epithelial cell line (hTERT-POMEC) (61), canine bronchiolar epithelial cell line (hTERT-CBECs) (77), and pig liver cell line (GalT-KO-hep) (82). So far, anchorage-independent growth, chromosomal abnormalities, and tumorigenic transformation have not been observed during the culture of these cell lines.

### Small mammalian and livestock cell lines

By comparing the establishment status of common small mammal (rats, mice, and rabbits) and livestock (such as pigs, cattle, and sheep) immortalized cell lines (Table 2), it is not difficult to find that most expression vectors carrying the *SV40-LT* or *hTERT* gene are transfected into cells to prolong their lifespan. Notably, the cell immortalization induced by the tetracycline Tet*-*on 3G system is reversible, and cell proliferation can be controlled with doxycycline (Dox), which is more flexible (93).

**Table 2:** Establishment of different cell lines in mammals.

**Species**	**Cell line**	**Establishment method**	**Immortality**	**References**
Rat	RKC2	*SV40-LT* was expressed in passaged kupffer cells	–	(83)
Mouse	EOE-2M and EOE-3M	Induced the expression of *HPV16 E6/E7* oncogene in primary enamel organ epithelial (EOE) dental cells	Maintained more than 30 generations	(84)
	FP5-1-3 cell line	Spontaneous generation from mammary buds in *p53*-null female embryos	–	(85)
	LmcMF	Introducted of *SV40-LT* into primary intestinal myofibroblasts.	At least 20 generations	(86)
	SmcMF	Spontaneous immortalized intestinal myofibroblasts	At least 20 generations	(86)
	AD-MSC	Knockout of *p53* gene in adipose-derived mesenchymal stem cells (MSCs)	Passaged more than 50 times	(87)
	Osteoblast cell line	Transfection of primary floxed Bmp2/4 mouse osteoblasts with *SV40-LT*	Grown more than 50 PDs	(27)
	Epithelial cell line	Embryonic mouse neuroepithelial cells were infected with a retrovirus containing the *c-myc* oncogene	–	(88)
Rabbit	Fibroblast cell line	Co-expression of mutant *CDK4, cyclin D1* and *hTERT* in fibroblasts	More than 11 generations	(89)
	Articular cartilage cell line	Transfected with plasmid encoding SV40 early functional gene	Up to 130 generations	(90)
	ImRMC	Induced lentivirus-mediated *SV40-LT* expression in primary melanocytes	–	(91)
	Epithelial cell line	Infection of primary corneal epithelial cells with recombinant *SV40*-adenovirus vector	Grown over hundreds of generations	(92)
Swine	Granulosa cell line	Conditionally expressed *SV40-LT* gene in primary granulosa cells using tetracycline-induced Tet-On 3G system	Stable proliferation for at least 6 months	(93)
	siNEC and siTEC	Transfer of *SV40-LT* into nasal and tracheal epithelial cells	Over 30 passages, the doubling time is cut in half	(94)
	Ttag and Puro	Transfer of lentiviral vector expressing *SV40-LT* into primary porcine spermatogonial stem cells	More than 35 passages	(95)
	GalT-KO-hep and WT	GalT-KO and wild-type pig primary hepatocytes were transfected with *SV40-LT* lentiviral vector	More than 20 generations	(82)
	Fibroblast cell line	Sleeping beauty transposon-mediated ectopic expression system of porcine *TERT*	Over 40 generations	(96)
	Endothelial cell line	Primary endothelial cells were transfected with plasmid pRNS-1 carrying neomycin resistance gene and *SV40-LT*	The doubling time was about 17.6 h	(80)
Cattle	Epithelial cell line	Mammary epithelial cells were infected by retrovirus with the *SV40-LT* plasmid	Up to 80 PDs in 10 months	(18)
	Epithelial cell line	Transfer of lentiviral vectors encoding *cyclin D1*, mutant *CDK4*, and *hTERT* genes into colon-derived epithelial cells	Over 15 generations	(97)
	Germ cell line	Constructed pEGFP-*c-myc* and pEGFP-*hTERT* expression vectors and transfected 5-month-old calf sperm stem cells	About 100 PDs in 140 days	(69)
	BMES	Muscular epithelial cell spontaneously immortalized	Cultured for 62 generations	(66)
Sheep	Endothelial cell line	*HPV16 E6/E7* open reading frames were permanently transfected into fifth generation fetal pulmonary artery endothelial cells	At least 28 passages	(98)
	mMTSV-54/93 and TIGEF	Transfection of plasmid DNA encoding *SV40-LT* gene into goat fibroblasts	Faster doubling time	(99)

Indicates that immortality is not mentioned in the citation in place of previous sentence.

As an example, the characteristics of immortalized pig cell lines separately obtained by transfecting *SV40-LT* and *hTERT* are compared (Table 3). It is observed that immortalization effects can be evaluated from the aspects of cell lifespan, telomerase activity, passage times, PDs, cell morphology, and tumorigenicity.

**Table 3:** Characteristics of immortalized pig cell lines established by transfecting *SV40-LT* and *hTERT*.

**Establishment method**	**Cell line**	**Characteristics**	**Immortality**	**References**
Transfection of *TERT* gene	Fibroblast cell line	It had anchorage dependency, and did not form any colonies on soft agar	Cultured for 30–45 passages	(60)
	hTERT-POMECs	No chromosome abnormality and tumorigenicity transformation	Cultured for more than 150 passages *in vitro*	(61)
	Fibroblast cell line	The cell line continued to grow after more than 40 passages, and *pTERT* maintained stable expression	Over 40 generations	(96)
	iPMSCs	With the ability to differentiate into neurons, cardiomyocytes, germ cells, and islet-like cells	More than 80 passages	(62)
	EE cell line	It had similar phenotypic and functional characteristics to the primary endocardial endothelium cells	Over 100 generations	(63)
	SUVECs	It had contact inhibition, serum demand and anchorage dependent growth	50 generations	(64)
Induced the expression of *SV40-LT*	Granulosa cell line	Able to reproduce stably for at least 6 months, with reduced cell proliferation following withdrawal from Dox	Stable proliferation for at least 6 months	(93)
	siNEC and siTEC	Retained the biological characteristics of primary epithelial cells and no abnormal chromosomes	Over 30 passages, the doubling time is cut in half	(94)
	Ttag and Puro	No morphological abnormalities	More than 35 passages within seven months	(95)
	GalT-KO-hep	Retained the characteristics of primary porcine hepatocytes. No tumorigenicity	More than 20 generations	(82)
	MSCs	Possessed higher proliferative capacity, shown no signs of senescence and displayed a common phenotype similar to primary MSCs	Serially passages more than 20–30 times	(38)
	Endothelial cell line	The original features of endothelial cells were preserved	The doubling time was about 17.6 h	(80)

Indicates that immortality is not mentioned in the citation in place of previous sentence.

### Establishment of poultry cell lines

We summarized poultry cell lines and their characteristics, including chickens, ducks, geese, and quails (Table 4). It was found that few immortalized cell lines were successfully established in poultry compared with mammals, and that the existing poultry cell lines were mainly obtained from tumor tissues; some chemical carcinogens or oncogenic viruses were used to immortalize specific types of bird cells, and some continuous cell lines were spontaneously generated. There are two points worth noting: (i) the preadipocyte lines “ICP1” and “ICP2” successfully established by transfection with the chicken telomerase reverse transcriptase (*chTERT*) have a high proliferation potential without malignant transformation after long-term culture, which provides a new idea and theoretical reference for the acquisition of other immortalized poultry cell lines (7), and (ii) during the whole process of establishing immortalized cell lines, specific antibiotics can be used to screen out positive cells expressing the *SV40-LT* gene or other target genes and then select a single transforming focus for subculture, which is not only simple but also safer (27).

**Table 4:** Existing poultry cell lines and their characteristics.

**Species**	**Cell Line**	**Characteristics**	**Immortality**	**References**
Chick	CSC-1-5	Spontaneous emergence, the fibroblast cell line had a high proliferative state, high homogeneity and the same genetic background, normal cell cycle distribution without tumorigenesis, and transformation	Stable passage over 3 months	(100)
	ICP1 and ICP2	Acquired by transducting *chTERT* alone or in combination with *chTR*. They showed fibroblast-like morphology without signs of malignant transformation, revealed high telomerase activity and retained adipocyte differentiation capacity	Cultured *in vitro* over 100 passages	(13)
	CEL-im	Spontaneous generation without oncogenic treatment, 0.8–1.1 PDs per day, and negative for telomerase activity	Cultured over 120 passages	(101)
	DF-1	Spontaneous emergence, they demonstrated a fibroblast-like morphology during culture, did not contain endogenous sequences associated with ASV or ALSV, and supported replication of avian retroviruses	–	(102)
	LMH	It obtained from liver tumor tissue after injecting diethylnitrosamine, had triploid karyotype and 6 marker chromosomes. After the 40th passage, the growth rate gradually increased and the cell morphology changed	Cultured 120 passages in 5 years	(103)
Duck	DEE cell line	It had good adhesion ability and proliferative activity, no tumorigenicity, and the doubling time was about 17.6 h	50 generations	(104)
	DEF-TA	Expressing *SV40-LT* (obtained after more than 8 rounds of puromycin selection), PDs number increased every 30 to 48 h, and maintain fibroblast morphology	Passaged more than 30 times	(39)
Goose	Epithelial cell line	Spontaneous formation with a cubic morphology and constant chromosomal characteristics, they could efficiently transfect some plasmids carrying avian virus reporter genes and did not transform into tumorigenic cells	Grown over 65 passages	(105)
Quail	QM l-4 and QM 6-8	Seven avian myogenic cell lines derived from the fibrosarcoma cell line QT6	–	(106)
	Myocardial cell line	It obtained by injection of MC29 virus carrying the *v-myc*, without morphological changes, showing decreased growth and enhanced differentiation	More than 60 passages in 6 months	(107)
	QT	Injected with 7,12-dimethylbenzylanthracene, MNNG and 3-methylcholanthrene (carcinogens) and isolated from tumor tissue. The fibrosarcoma cell line had undergone ~10 passages and was characteristic by tumorigenic transformation	Undergone ~10 passages	(108)
	Cartilage cell line	Acquired by infection with MC29, it stimulates chondrocyte proliferation and progressively reduces doubling time	About 70 generations in 16 months	(109)

Indicates that immortality is not mentioned in the citation in place of previous sentence.

## Conclusions

Establishing an ideal immortalized cell line with infinite proliferation ability and maintaining the characteristics of its source tissue cells cannot only avoid the complicated process of primary cell separation and purification, reduce the time and energy consumption of researchers, and save the cost of experiments, it is also conducive to the research on scientific issues such as gene function of livestock and poultry, and rapidly promotes the development of science. Since immortalized cells can be passaged multiple times *in vitro*, researchers can immortalize cells that are difficult to passage, slow to proliferate, and prone to senescence, and provide more cell resources for related experiments. Nevertheless, whether the functional cells from different species adopt the same immortalization method, and how to quickly and efficiently prepare immortalized cells and to ensure the immortalized cells maintaining the original characteristics have not yet been solved and require more in-depth research. In summary, the application of immortalized cells has broad prospects. The continuous improvement of immortalized cell line establishment technology is conducive to further research in molecular biology and other scientific fields.

## Author contributions

DG participated in literature collection, drafted the manuscript, and revised it. LZ participated in the design of this review and revised it. XW participated in literature collection. JZ helped draft the manuscript. SL conceived the review, participated in literature collection, revised the manuscript, and finally agreed to publish it. All authors read and approved the final version of the manuscript.

## Funding

This research was funded by the National Natural Science Foundation of China (Grant No. 31972550), Guangdong Province (Grant Nos. 2020A1515011576 and 2020B1515420008), and College of Coastal Agricultural Sciences—Ph.D. Start-up Fee and Postgraduate Training Fund (Grant No. 060302052104).

## Conflict of interest

The authors declare that the research was conducted in the absence of any commercial or financial relationships that could be construed as a potential conflict of interest.

## Publisher's note

All claims expressed in this article are solely those of the authors and do not necessarily represent those of their affiliated organizations, or those of the publisher, the editors and the reviewers. Any product that may be evaluated in this article, or claim that may be made by its manufacturer, is not guaranteed or endorsed by the publisher.

## Abbreviations

PBS: Phosphate buffered solution
FBS: fetal bovine serum
PDs: population doublings
MNNG: N-methyl-N-nitro-N-nitrosoguanidine
SV40-LT: Simian virus 40 large T antigen
HPV E6/E7: human papilloma virus E6 or E7 protein
EBV: Epstein-Barr virus
*c-myc*: cellular-myelocytomatosis viral oncogene
hTERT: human telomerase reverse transcriptase
TR: telomerase RNA
PAI: plasminogen activator inhibitor
iPMSCs: immortalized porcine mesenchymal stem cells
EE: endocardial endothelium
SUVECs: swine umbilical vein endothelial cells
AEC: alveolar epithelial cell
BME: bovine microvascular endothelium
STCs: sheep trophoblast cells
bMECs: bovine mammary epithelial cells
Bag-1: Bcl-2-associated athanogene 1
TRPS-1: transcriptional repressor 1
CDKN2A: cyclin-dependent kinase inhibitor 2A
Dox: doxycyline
RKCs: rat Kupffer cells
EOE: enamel organ epithelial
MSCs: mesenchymal stem cells
CDK4: cyclin-dependent kinase 4
chTERT: chicken telomerase reverse transcriptase
ASV: avian sarcoma virus
ALSV: avian leukosis sarcoma virus
DEFs: duck embryo fibroblasts.
